# Mesenchymal stem cells in the treatment of traumatic articular cartilage defects: a comprehensive review

**DOI:** 10.1186/s13075-014-0432-1

**Published:** 2014-09-26

**Authors:** Troy D Bornes, Adetola B Adesida, Nadr M Jomha

**Affiliations:** Department of Surgery, University of Alberta, Laboratory of Stem Cell Biology and Orthopaedic Tissue Engineering, Edmonton, Alberta T6G 2E1 Canada; Division of Orthopaedic Surgery, Department of Surgery, University of Alberta, Edmonton, Alberta T6G 2B7 Canada

## Abstract

Articular cartilage has a limited capacity to repair following injury. Early intervention is required to prevent progression of focal traumatic chondral and osteochondral defects to advanced cartilage degeneration and osteoarthritis. Novel cell-based tissue engineering techniques have been proposed with the goal of resurfacing defects with bioengineered tissue that recapitulates the properties of hyaline cartilage and integrates into native tissue. Transplantation of mesenchymal stem cells (MSCs) is a promising strategy given the high proliferative capacity of MSCs and their potential to differentiate into cartilage-producing cells - chondrocytes. MSCs are historically harvested through bone marrow aspiration, which does not require invasive surgical intervention or cartilage extraction from other sites as required by other cell-based strategies. Biomaterial matrices are commonly used in conjunction with MSCs to aid cell delivery and support chondrogenic differentiation, functional extracellular matrix formation and three-dimensional tissue development. A number of specific transplantation protocols have successfully resurfaced articular cartilage in animals and humans to date. In the clinical literature, MSC-seeded scaffolds have filled a majority of defects with integrated hyaline-like cartilage repair tissue based on arthroscopic, histologic and imaging assessment. Positive functional outcomes have been reported at 12 to 48 months post-implantation, but future work is required to assess long-term outcomes with respect to other treatment modalities. Despite relatively positive outcomes, further investigation is required to establish a consensus on techniques for treatment of chondral and osteochondral defects with respect to cell source, isolation and expansion, implantation density, *in vitro* precultivation, and scaffold composition. This will allow for further optimization of MSC proliferation, chondrogenic differentiation, bioengineered cartilage integration, and clinical outcome.

**Electronic supplementary material:**

The online version of this article (doi:10.1186/s13075-014-0432-1) contains supplementary material, which is available to authorized users.

## Introduction

Articular cartilage (AC) injury following joint trauma is a major risk factor for the development of osteoarthritis (OA), a condition that results in significant patient morbidity and substantial cost to healthcare systems [1–4]. Approximately 10 to 25% of the population suffers from OA, with increased prevalence noted in older age groups [4,5]. OA is irreversible and eventually requires joint replacement for alleviation of pain and restoration of function as it progresses to end-stage disease. Due to the limited capacity of AC to repair, early intervention is required to prevent progression to OA [6]. Effective management options are limited at present, resulting in a drive to develop novel tissue engineering techniques to resurface AC defects [7].

Current treatment modalities aim to restore AC through primary repair, stimulation of adjacent tissue and graft implantation. Primary repair involves rigid fixation of osteochondral fractures in an acute setting [8]. Microfracture and subchondral drilling breach subchondral bone to allow migration of cells and chemical mediators into defects [6]. Although this leads to defect filling with repair tissue that is predominantly fibrocartilage [9], reasonable results can be obtained in the short- to intermediate-term with proper rehabilitation [10,11].

Osteochondral autologous transplantation and mosaicplasty are performed through transplanting one or more osteochondral autografts from healthy, non-weight-bearing surfaces [12]. Although intermediate-term outcomes have been positive, outcomes are variable over longer periods of time [12,13]. Furthermore, donor site morbidity is the major downside of this technique [13]. Allogeneic transplantation is an alternative strategy that allows for resurfacing of large osteochondral defects. Fresh allografts stored at 4°C provide good clinical outcomes [14], but are logistically difficult given the need for donor-recipient size matching, testing for infectious diseases and implantation within a short time frame to ensure chondrocyte viability [15]. Freezing of tissue allows for longer-term storage, but outcomes deteriorate quickly following implantation of frozen allografts [16]. However, cryopreservation could be a suitable alternative in the future given the establishment of vitrification protocols that have yielded promising results [17]. Bioengineered scaffolds implanted alone, or in conjunction with marrow stimulation in autologous matrix-induced chondrogenesis, effectively fill joint defects and improve function, but it is currently unclear whether the resulting repair tissue recapitulates the properties of AC [18,19].

Autologous chondrocyte implantation (ACI) involves chondrocyte isolation from cartilage in non-weight bearing areas, expansion *ex vivo* and re-implantation into the cartilage defect covered by a periosteal graft [20]. In matrix-associated ACI (MACI), chondrocytes are implanted on three-dimensional porous scaffolds that facilitate three-dimensional repair tissue formation and defect filling [11]. Positive outcomes have been reported at 7 to 13 years for knee lesions [11,20], and 2 to 5 years for ankle lesions [21,22]. However, both techniques require two invasive surgical procedures [20]. Low chondrocyte yield, loss of capacity to make hyaline cartilage-like extracellular matrix (ECM) due to chondrocyte de-differentiation, and chondrocyte senescence are concerns [23–25].

Transplantation of mesenchymal stem cells (MSCs) is a cell-based strategy that has the potential to resurface AC defects while avoiding the downsides of ACI/MACI. MSCs have an enhanced proliferative capacity and can be reproducibly differentiated into chondrocytes [26]. Cell harvesting does not require an invasive procedure or wounding of AC at another site.

The aim of this article is to provide a comprehensive review of MSC-based cartilage regeneration from bench to bedside and a discussion of the current technical considerations in MSC transplantation for treatment of traumatic, focal chondral and osteochondral defects.

## Methods

**Figure 1:**
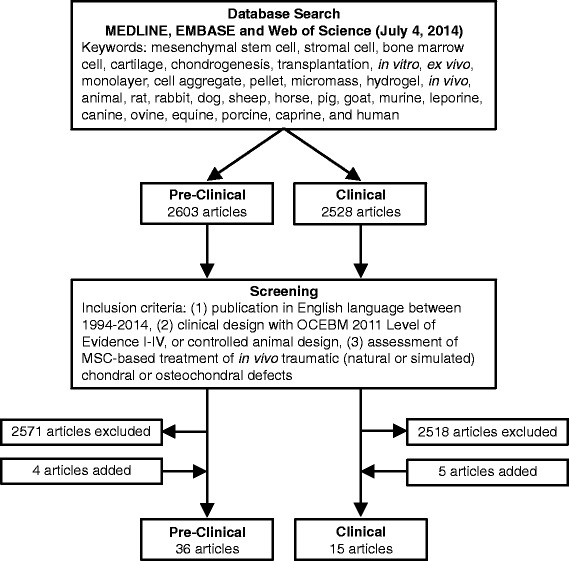
**Search strategy and selection of pre-clinical and clinical literature.** MSC, mesenchymal stem cell; OCEBM, Oxford Centre for Evidence-Based Medicine.

A comprehensive literature search was performed of MEDLINE, EMBASE and Web of Science databases to identify English articles published between 1994 and 2014 using various combinations of the following keywords: mesenchymal stem cell, stromal cell, bone marrow cell, cartilage, chondrogenesis, transplantation, *in vitro*, *ex vivo*, monolayer, cell aggregate, pellet, micromass, hydrogel, explant, *in vivo*, animal, rat, rabbit, dog, sheep, horse, pig, goat, murine, leporine, canine, ovine, equine, porcine, caprine, and human. Search steps performed within each database specifically for *in vitro*, *in vivo* animal and clinical literature are detailed in Additional file 1. Compilation of database outputs produced 6,137, 2,603 and 2,528 publications, respectively, for these searches. *In vivo* articles were then screened and included if they met the following criteria: (1) publication in English between 1994 and 2014; (2) clinical design with Oxford Centre for Evidence-Based Medicine 2011 level of evidence I to IV [27] or controlled animal design; and (3) assessment of MSC-based treatment of *in vivo* traumatic (natural or simulated), focal chondral or osteochondral defects. Relevant articles found within reference lists and within the journal *Cartilage* were also screened and considered for inclusion. This process yielded 36 pre-clinical *in vivo* animal studies, including 21 small animal and 15 large animal studies, and 15 clinical studies (Figure 1). Only key *in vitro* articles were included in our review as several hundred relevant articles were found within our initial search.

## Mesenchymal stem cells

MSCs are spindle-shaped cells capable of rapid proliferation and self-renewal contained within a number of tissues, including bone marrow, synovial tissue, blood, adipose tissue, and periosteum [26,28,29]. Their multi-lineage potential allows for differentiation into a variety of cell types in order to create and repair mesenchymal tissues. MSCs have been differentiated into chondrogenic, osteogenic, and adipogenic pathways [26]. Mediators capable of promoting MSC chondrogenesis, such as transforming growth factor-beta (TGF-β) and dexamethasone, have been elucidated using simplified *in vitro* models [30].

## Mesenchymal stem cell-based cartilage regeneration from bench to bedside

### *In vitro* studies

MSC chondrogenesis can be induced within simple *in vitro* models consisting of cell monolayers or cell aggregates, pellets, micromasses, or transwell cultures containing multiple layers of cells and ECM [26,31–33]. High-density aggregation was achieved through the use of centrifugation and situates cells in a three-dimensional environment that fosters cellular interaction, mimicking cell condensation of mesenchymal cells during embryonic cartilage development and chondrogenic ECM formation [26,30]. Alternatively, various biomaterials have been used as matrices on which MSCs are differentiated. MSCs embedded within collagen, agarose, alginate, chitosan and hyaluronic acid (HA) gels form aggregates of tissue that contain chondrocytes and cartilaginous ECM [34–38]. MSC-seeded porous scaffolds composed of collagen, HA, silk, decellularized cartilage ECM, polyglycolic acid (PGA), polylactic acid (PLA) and polylacticglycolic acid (PLGA) create tissue that histologically resembles hyaline cartilage [39–43].

### *Ex vivo* studies

Chondral and osteochondral explant models allow for cartilage repair tissue formation to be assessed within simulated defects in controlled *in vitro* environments. Porcine MSCs embedded in agarose gel implanted within chondral explants showed an abundance of type II collagen and glycosaminoglycan (GAG) matrix after 6 weeks of culture [35]. Similarly, human MSCs embedded in alginate gel and implanted within osteochondral explants for 4 weeks had collagen II gene expression and GAG production consistent with hyaline cartilage [34]. MSC-seeded gels displayed minimal integration with surrounding explant cartilage after 6 weeks of culture [35]. This may be due, in part, to the absence of sufficient remodeling time or *in vivo* factors, such as mechanical stimulation, required for integration to occur [44].

### *In vivo* animal studies

Animal models have provided pre-clinical *in vivo* assessment of MSCs in the treatment of AC defects. Starting with the work of Wakitani and colleagues in 1994 [7], MSC-based techniques have yielded positive outcomes in regenerating AC in several small animal studies involving rabbits [45–62] and rats [63,64]. Various MSC injection and transplantation protocols have been used to treat simulated, focal chondral and osteochondral defects in large animals such as sheep, goats, pigs, and horses [38,65–78]. These large animal studies are summarized in Table 1.

**Table 1:** **Large animal studies assessing mesenchymal stem cell-based treatment of chondral and osteochondral defects**

**Scientific publication**	**Animal model**	**Simulated defect characteristics**	**Implanted/injected construct**	**Follow-up period**	**Key findings**
Guo *et al*. (2004) [65]	28 sheep	Medial femoral condyle osteochondral defects; cylindrical (8 mm diameter)	Implantation of isolated BM-derived MSCs seeded on a TCP scaffold; compared to cell-free scaffolds and empty defects	6 months	Macroscopic: smooth, integrated tissue in MSC group. Histologic: proteoglycan and type II collagen consistent with hyaline cartilage in MSC group, compared with fibrocartilage in cell-free group; subchondral osseous regeneration. Biochemical: GAG quantity in MSC group was 89% of native cartilage
Wayne *et al*. (2005) [66]	10 dogs	Medial and lateral femoral condyle osteochondral defects; cylindrical (6 mm diameter)	Implantation of isolated BM-derived MSCs suspended in alginate and seeded on a PLA scaffold; precultivated for 3 wk; compared to cell-free scaffolds	1.5 months	Macroscopic: improved coverage of defects in MSC group. Histologic: mixture of hyaline and fibrocartilage integrated with surrounding tissue; higher quality tissue in MSC group compared with cell-free group; no mineralization noted within osseous defects. Mechanical: lower resistance to compression than native cartilage
Ando *et al.* (2007) [67]	9 piglets	Medial femoral condyle chondral defects; cylindrical (8.5 mm diameter)	Implantation of isolated, allogeneic synovial tissue MSCs derived from piglets and cultured in a three-dimensional scaffold-free TEC; compared to empty defects	6 months	Macroscopic: greater defect coverage in TEC group; subchondral erosion in the empty defects. Histologic: smooth, integrated tissue containing proteoglycans and type II collagen in the TEC group; empty defects showed signs of OA; higher ICRS scores in the TEC group. Mechanical: similar viscoelastic properties between TEC and native cartilage
Lee *et al.* (2007) [68]	27 mini-pigs	Medial femoral condyle chondral defects; cylindrical (8.5 mm diameter)	Injection of isolated BM-derived MSCs with HA (Synvisc) followed by HA weekly × 2 wk; compared to HA alone	3 months	Macroscopic: greater defect coverage in the MSC + HA group. Histologic: hyaline-like cartilage noted in MSC + HA group; minimal defect filling in HA group; improvement in Wakitani histologic score with MSCs
Saw *et al.* (2009) [69]	15 goats	Femoral trochlea chondral defects; cylindrical (4 mm diameter)	Injection of BMDC collection with HA (Hyalgan) weekly for 3 wk starting 1 wk after subchondral drilling; compared to drilling with or without HA	6 months	Macroscopic: greater defect coverage in the BMDC + HA group. Histologic: HA group had some proteoglycans and type II collagen mixed with type I collagen; BMDC + HA group had superior proteoglycan and type II collagen content; cell morphology was improved in the BMDC + HA group
Zscharnack *et al.* (2010) [38]	10 sheep	Medial femoral condyle osteochondral defects; cylindrical (7 mm diameter)	Implantation of isolated BM-derived MSCs in type I (rat) collagen gel either immediately following seeding or after 2 wk of precultivation	6 months	Macroscopic: precultivation group produced more homogenous hyaline-like cartilage. Histologic: significantly better O’Driscoll and ICRS scores in the precultivation group compared with non-precultivated group, specifically with respect to surface features, integration, cell distribution, and mineralization. Mechanical: precultivated tissue was firm
Shimomura *et al.* (2010) [70]	7 pigs, 6 piglets	Medial femoral condyle chondral defects; cylindrical (8.5 mm diameter)	Implantation of isolated synovial tissue MSCs derived from piglets and cultured in a three-dimensional scaffold-free TEC; compared to empty defects	6 months	Macroscopic: greater defect coverage in TEC group. Histologic: good integration of tissue that stained well for proteoglycans in the TEC group versus signs of OA in empty defects; higher ICRS scores in the TEC group. Mechanical: similar properties between TEC and native tissue
Wegener *et al.* (2010) [71]	9 sheep	Medial femoral condyle chondral defects; cylindrical (8 mm diameter)	Implantation of BM cells in fibrin glue seeded on a PGA scaffold; secured to subchondral bone by PLGA darts; compared to cell-free scaffolds	3 months	Macroscopic: BM-seeded scaffolds had improved regeneration compared with cell-free scaffolds. Histologic: variation noted with fibrous tissue in some and hyaline-like cartilage in other BM cell-seeded scaffolds; O’Driscoll score was similar between cell-free and cell-seeded scaffolds
Marquass *et al.* (2011) [72]	9 sheep	Medial femoral condyle osteochondral defects; cylindrical (7 mm diameter)	Implantation of isolated BM-derived MSCs in type I (rat) collagen gel implanted either immediately following seeding or after 2 wk of precultivation; compared to MACI	12 months	Macroscopic/histologic: significantly better O’Driscoll and ICRS scores with precultivated MSCs compared with both non-precultivated MSCs and MACI, specifically with respect to surface quality, matrix quality and integration; type II collagen content was superior in precultivated group. MRI: precultivated MSCs were similar to MACI but significantly better than non-precultivated MSCs on the MOCART score
McIlwraith *et al.* (2011) [73]	10 horses	Medial femoral condyle chondral defects (1 cm^2^)	Injection of isolated BM-derived MSCs with HA (Hyvisc) into the knee joint 1 month after MFX; compared to cell-free HA injection and MFX	12 months	Macroscopic: greater repair tissue area with MSCs, but no difference in volume. Histologic: no difference in surface, structure, integration, cellular architecture, and subchondral regeneration; contradictory proteoglycan and aggrecan staining. Biochemical: equivalent GAG. Mechanical: tissue derived from MSCs was firmer. MRI: no difference
Ando *et al.* (2012) [74]	6 piglets	Medial femoral condyle chondral defects; cylindrical (8.5 mm diameter)	Implantation of isolated, allogeneic synovial MSCs and cultured in a three-dimensional scaffold-free TEC; compared to empty defects	6 months	Histologic: tissue containing proteoglycans in the TEC group; empty defects were partially covered with fibrous tissue and showed signs of OA; higher O’Driscoll scores in the TEC group. Mechanical: similar properties between TEC and native cartilage
Zhang *et al.* (2012) [75]	20 mini-pigs	Femoral trochlea chondral defects; cylindrical (6 mm diameter)	Implantation of BMDCs or isolated, expanded BM-derived MSCs in type II collagen (porcine) hydrogel; compared to cell-free gels	2 months	Macroscopic: good defect filling with both MSCs and BMDCs; irregularity with cell-free gels. Histologic: hyaline-like cartilage with both MSCs and BMDCs; O’Driscoll score was greater in the MSC group at 4 wk, but equivalent between the BMDC and MSC groups at 8 wk
Bekkers *et al.* (2013) [76]	8 goats	Medial femoral condyle chondral defects; cylindrical (5 mm diameter)	Implantation of chondrons and BM-derived MSCs suspended in fibrin glue; compared to MFX	6 months	Macroscopic: improved defect filling with MSC + chondrons in comparison to MFX. Histologic: O’Driscoll score was significantly higher in the MSC + chondron group. Biochemical: GAG content and GAG/DNA in the repair tissue was greater in the MSC + chondron group than the MFX group
Kamei *et al.* (2013) [77]	16 mini-pigs	Patella chondral defects; cylindrical (6 mm diameter)	Magnetic accumulation of injected ferumoxide labeled MSCs; compared to gravity-focused MSCs	3 months	Arthroscopic: improved smoothness and integration with magnetic accumulation. Histologic: superior integration and type II collagen content with magnetic accumulation; improved scoring on the Wakitani scale
Nam *et al.* (2013) [78]	18 goats	Medial femoral condyle chondral defects; cylindrical (5 mm diameter)	Injection of isolated BM-derived MSCs weekly (×3 wk) starting 2 wk after subchondral drilling; compared to drilling alone	6 months	Macroscopic: smooth, integrated tissue with MSCs versus partial, irregular filling with drilling alone. Histologic: O’Driscoll score was significantly higher in the MSC group; improved proteoglycan and type II collagen content with MSCs. Biochemical: higher GAG quantity with MSCs

BM, bone marrow; BMDC, bone marrow-derived cell; GAG, glycosaminoglycan; HA, hyaluronic acid; ICRS, International Cartilage Repair Society; MACI, matrix-associated autologous chondrocyte implantation; MFX, microfracture; MOCART, Magnetic Resonance Observation of Cartilage Repair Tissue; MRI, magnetic resonance imaging; MSC, mesenchymal stem cell; OA, osteoarthritis; PGA, polyglycolic acid; PLA, polylactic acid; PLGA, polylactide co-glycolide; TCP, tricalcium phosphate; TEC, tissue-engineered construct; wk, week(s).

Intra-articular injection of MSCs into rabbit knees containing femoral trochlea osteochondral defects led to resurfacing with fibrous tissue that failed to remodel into hyaline cartilage over 24 weeks [52]. In contrast, MSCs implanted directly into the defect site produced cartilage-like tissue that remodeled with time to produce both cartilage and bone components similar to surrounding native osteochondral tissue. In another study, MSCs injected in conjunction with HA into porcine knees with partial-thickness chondral defects led to good defect coverage with hyaline-like cartilage at 12 weeks post-injection [68]. HA alone produced minimal defect filling in this time frame.

Other groups have performed MSC injection in association with subchondral drilling or microfracture [69,73,78]. Serial MSC injections performed weekly for 3 weeks after subchondral drilling for treatment of simulated chondral defects within the distal femur of goats produced integrated repair tissue consistent with hyaline cartilage after 6 months [78]. In a similar model, Saw and colleagues [69] used bone marrow aspirate cell collections injected weekly with HA. They found improved content of proteoglycan and type II collagen within femoral trochlea chondral defects that received cell injection in comparison to those that received only HA. In contrast to these findings, McIlwraith and colleagues [73] found no difference between HA and an HA-MSC combination in several histologic parameters, magnetic resonance imaging (MRI) evaluation, and GAG quantity at 1 year in horses that received an injection and microfracture. Possible reasons for inconsistent outcomes include variation in the number of injections, length of follow-up and species.

One potential drawback of intra-articular injection involves cell dispersion, associated lack of focus of injected contents into a defect site, and the potential for an insufficient amount of seeded cells required for regeneration. The use of magnetic labeling of cells and an external magnet has been proposed as a minimally invasive method to deliver injected MSCs to defects. In mini-pig knees, ferumoxide-labeled MSCs were directed over patella chondral lesions by magnet for 10 minutes following injection and produced superior arthroscopic and histologic scores to an injection directed by gravity [77].

Various constructs for implantation have been proposed in the pre-clinical literature. A scaffold-free, three-dimensional tissue-engineered construct (TEC) derived from monolayers containing differentiated MSCs has been investigated [70,74]. Over 6 months, TECs implanted within porcine femoral condyle chondral defects created repair tissue with a superficial fibrous layer and deep AC-like layer [70].

Transplantation of MSC-seeded matrices composed of collagen, PLA, PGA, PLGA, polycaprolactone, fibrin, chitosan, alginate, silk, demineralized bone matrix, and tricalcium phosphate was successfully performed in several other small and large animal studies [7,45–49,51,54,56,57,59–61,63,71,79]. Defect resurfacing with hyaline-like cartilage tissue was reported in the majority of cases at 4 to 24 weeks post-implantation with more integrated, mature tissue found at later time points. Some groups also noted the presence of bone regeneration within the osseous component of osteochondral defects [47,48,57,61,65].

Implantation of matrices seeded with MSCs that were precultivated *in vitro* for 2 to 3 weeks prior to implantation is an alternative protocol that has been assessed in three other studies [38,66,72]. Zscharnack and colleagues [38] showed that precultivated MSC-seeded collagen gels implanted within sheep osteochondral defects produced superior repair tissue to non-precultivated MSC-seeded gels based on International Cartilage Repair Society (ICRS) histologic scoring at 6 months post-implantation. Marquass and colleagues [72] had similar findings after 1 year and also showed that precultivated MSCs had better histologic outcomes than precultivated chondrocytes (MACI).

Bone marrow nucleated cells - often described as bone marrow-derived cells (BMDCs) in the literature [80,81] - were seeded on collagen gels and compared with isolated, expanded MSCs by Zhang and colleagues [75]. After 2 months, both cell types produced histologically and macroscopically equivalent hyaline-like cartilage repair tissue within porcine femoral trochlea chondral defects.

Co-transplantation of chondrons and MSCs suspended within fibrin glue into goat femoral condyle chondral defects was assessed in another study [76]. This technique showed superior defect filling, O’Driscoll histologic scoring and biochemical GAG quantity in comparison to microfracture. However, co-transplantation was not compared with MSC or chondrocyte transplantation alone.

Animal studies have yielded positive pre-clinical results that have provided support and direction for MSC-based therapies in humans. Specific techniques such as MSC injection, and transplantation of both isolated MSCs and BMDCs have been taken into the clinical realm. Other techniques such as scaffold-free TEC, magnetically guided MSC injection, co-transplantation, and MSC precultivation have only been reported in the animal literature to date.

### Clinical studies

A growing body of clinical evidence supports MSC implantation as an effective treatment for traumatic AC injury (Table 2). Cells derived from autologous bone marrow aspirates from the iliac crest have been used for treatment of focal, traumatic chondral and osteochondral defects of the femoral condyle [81–87], femoral trochlea [84,88,89], talus [80,90,91], tibial plateau [89], and patella [84,88,89]. Other studies have addressed the use of this technique in managing other defect types, such as osteochondral lesions arising from osteochondritis dissecans [92,93], septic arthritis [94] and unicompartmental OA [95].

**Table 2:** **Clinical studies assessing mesenchymal stem cell-based treatment of traumatic chondral and osteochondral defects**

**Scientific publication**	**Study type**	**Subject details**	**Defect characteristics**	**Implanted/injected construct**	**Follow-up period**	**Key findings**
Kuroda *et al*. (2007) [82]	Case report: level IV evidence	1 M (age 31 y)	1 medial femoral condyle chondral defect (6.0 cm^2^) from trauma	Implantation of isolated BM-derived MSCs within porcine type I collagen gel on a collagen scaffold; covered by a periosteal flap	12 months	Arthroscopic: firm, smooth repair tissue. Histologic: hyaline-like cartilage covered superficially by fibrous tissue. MRI: focal chondral and subchondral irregularities. Clinical: return to previous level of activity
Wakitani *et al*. (2007) [88]	Case series: level IV evidence	3: 2 M, 1 F (age 32–45 y)	5 femoral trochlea (0.7-4.2 cm^2^) and 4 patella chondral defects (1.0-1.7 cm^2^); defects in 2/3 participants from trauma	Implantation of isolated BM-derived MSCs within bovine type I collagen gel on a porcine collagen scaffold; covered by a periosteal flap or synovium; adjunctive subchondral drilling	18 months	Arthroscopic: firm, smooth tissue. Histologic: atypical cartilage. MRI: complete coverage of defects but quality unclear. Clinical: improvement of symptoms and return to work; IKDC improvement
Giannini *et al*. (2009) [80]	Case series: level IV evidence	48: 27 M, 21 F (mean age 28.5 ± 9.5 y)	48 talar dome osteochondral defects (2.07 ± 0.48 cm^2^); 35 from trauma; previous MFX, debridement or ACI in 15	Implantation of BMDCs suspended within collagen/platelet paste or seeded on HA (Hyaff-11) scaffold	24-35 months	Arthroscopic: smooth tissue in some, hypertrophic in others; all integrated with firmness of native cartilage. Histologic: mixed with some hyaline quality. MRI: newly formed tissue in all lesions. Clinical: improvement in AOFAS scores with time and return to sports with no difference between scaffold types; worse outcomes with previous surgery
Buda *et al*. (2010) [81]	Case series: level IV evidence	20: 12 M, 8 F (mean age 28.5 ± 9.5 y)	16 medial femoral condyle and 6 lateral condyle osteochondral defects (no area provided); 18 traumatic and 2 OCD defects	Implantation of BMDCs seeded on a HA (Hyalofast) scaffold supplemented with platelet-rich fibrin; adjunctive meniscus repair or debridement, ACL-R, or HTO	29 ± 4.1 months	Histologic: collagen II noted throughout repair tissue with focal proteoglycan content consistent with hyaline-like cartilage. MRI: variable signal intensity that correlated with KOOS score. Clinical: improvement in IKDC and KOOS scores post-operatively
Giannini *et al*. (2010) [90]	Prospective comparative study: level III evidence	81: 47 M, 34 F (mean age 30 ± 8 y); 25 BMDC; 10 ACI; 46 MACI	81 talar dome osteochondral defects (>1.5 cm^2^) from trauma	Implantation of BMDCs seeded on a HA (Hyaff-11) scaffold supplemented with platelet-rich fibrin	59.5 ± 26.5 months	Arthroscopic: good defect coverage. Histologic: hyaline-like cartilage noted. MRI: complete integration in 76% and homogenous tissue in 82% of all cases with hypertrophy in 3 BMDC and 2 ACI patients. Clinical: improvement in AOFAS scores after surgery with no difference between BMDC-scaffold implants, ACI and MACI; lower overall cost for BMDC transplantation compared to ACI/MACI
Haleem *et al*. (2010) [83]	Case series: level IV evidence	5: 4 M, 1 F (mean age 25.4 y)	5 femoral condyle chondral defects (3–12 cm^2^); 2 traumatic, and 3 OCD defects (1 OA from neglected OCD)	Implantation of isolated BM-derived MSCs within platelet-rich fibrin glue; covered by a periosteal flap	12 months	Arthroscopic: smooth tissue. MRI: complete defect filling with good congruity in 3/5 patients. Clinical: improvement in Lysholm and RHSSK scores with return to sports; worse outcomes in 1 patient with pre-operative OA
Nejadnik *et al*. (2010) [84]	Prospective comparative study: level III evidence	72: 38 M, 34 F (mean age 44.0 ± 11.4 y), 36 MSCs; 36 ACI	13 patella, 4 femoral trochlea, 12 femoral condyle, and 7 multiple knee chondral defects (4.6 ± 3.5 cm^2^); 14 traumatic, 20 OA and 2 other defects	Implantation of isolated BM-derived MSCs; covered by a periosteal flap; adjunctive partial meniscectomy, patellar realignment, ACL-R, or HTO	24 months	Arthroscopic: smooth tissue in most cases. Histologic: aggrecan and collagen II content consistent with hyaline cartilage. Clinical: greater improvement in SF-36 Physical Role Functioning in MSCs versus chondrocytes; equivalent IKDC, Tegner and Lysholm score improvement following both MSC and chondrocyte transplantation; superior outcomes in males versus females
Gobbi *et al*. (2011) [89]	Case series: level IV evidence	15: 10 M, 5 F (mean age 48 y, range 32–58 y)	7 patella, 6 femoral trochlea, 4 medial tibial plateau, 6 medial femoral condyle, and 1 lateral condyle chondral defects (9.2 ± 6.3 cm^2^); all defects from trauma; 6 patients had multiple defects	Implantation of BMDCs mixed with batroxobin (Plateltex Act) to produce a clot; covered by a type I/III collagen matrix (Chondro-Gide); adjunctive ACL-R, HTO, patellar realignment	24-38 months	Arthroscopic: smooth, integrated tissue in all cases; no hypertrophy. Histologic: variability with properties of hyaline and fibrocartilage. MRI: complete defect filling in 80%, integration in 93%, and no hypertrophy in all patients. Clinical: improvement in all scores (VAS, KOOS, Tegner, Marx, IKDC and Lysholm) following surgery; patients with single lesions and smaller lesions had better outcomes
Kasemkijwa-ttana *et al*. (2011) [85]	Case series: level IV evidence	2 M (age 24–25 y)	2 lateral femoral condyle chondral (2.2-2.5 cm^2^)	Implantation of isolated BM-derived MSCs seeded on a type I collagen scaffold supplemented with fibrin glue; covered by a periosteal flap; adjunctive ACL-R, meniscal repair	30-31 months	Arthroscopic: good defect fill, integration and firmness. Clinical: significant improvement in IKDC score and KOOS post-operatively
Saw *et al.* (2011) [98]	Case series: level IV evidence	5: 1 M, 4 F (mean age 39.4 y, range 19–52 y)	3 focal defects: 1 lateral femoral condyle (2 cm^2^), 1 patella (8.8 cm^2^), 1 femoral trochlea (0.5 cm^2^); 2 OA defects	Injection of peripheral blood-derived MSCs with HA weekly (×5) starting 1 wk after subchondral drilling; adjunctive HTO or lateral patellar release; pre-injection GCSF	10-26 months	Arthroscopic: good filling in focal defects; range from devoid areas to smooth repair tissue in OA defects. Histologic: intense proteoglycan staining; type I collagen in superficial area with predominance to type II collagen in deep area; chondrocytes in subchondral drill holes
Gigante *et al.* (2012) [86]	Case report: level IV evidence	1 M (age 37 y)	1 medial femoral condyle chondral defect (3 cm^2^) from trauma	Implantation of BMDCs within fibrin glue (Tisseel) and coverage with a collagen membrane (MeRG) after arthroscopic MFX (CMBMC)	24 months	MRI: good defect filling with tissue that was isointense relative to native cartilage; no signs of bone edema. Clinical: return to activity and asymptomatic
Enea *et al*. (2013) [87]	Case series: level IV evidence	9: 5 M, 4 F (mean age 48 ± 9 y)	6 medial femoral condyle and 3 lateral condyle chondral defects (2.6 ± 0.5 cm^2^); previous meniscectomy, debridement or ACL-R	Implantation of BMDCs within fibrin glue and coverage with a PGA-HA membrane (Chondro-tissue) after arthroscopic MFX (CMBMC); adjunctive meniscectomy, osteochondral fixation, or trochlea resurfacing	22 ± 2 months	Arthroscopic: 1 normal, 3 nearly normal and 1 abnormal on ICRS CRA. Histologic: hyaline-like cartilage repair tissue. MRI: complete defect filling in all; mild subchondral irregularities in all; hypertrophy in 1 patient. Clinical: improvement in IKDC and Lysholm scores compared with pre-operative scores; no change in Tegner score from pre-injury; one failure
Giannini *et al*. (2013) [91]	Case series: level IV evidence	49: 27 M, 22 F (mean age 28.1 ± 9.5 y)	49 talar dome osteochondral defects (2.2 ± 1.2 cm^2^); 36 traumatic defects with unknown etiology in others; previous debridement, MFX, ACI, or BMDCs in 17	Implantation of BMDCs within collagen/platelet paste or seeded on HA (Hyaff-11) scaffold supplemented with platelet gel	48 months	MRI: complete defect filling in 45%, hypertrophy in 45%, integration in 65%, subchondral disruption in 65% of cases; 78% of repair area had hyaline quality. Clinical: improvement in AOFAS scores - maximal value at 24 months; decreased at 36–48 months; decreased AOFAS associated with fibrocartilage quality; return to pre-injury sports in 78%
Saw *et al*. (2013) [29]	RCT: level II evidence	49: 17 M, 32 F (mean age 38 ± 7 y); 25 MSC + HA; 24 HA	49 chondral defects of the knee (57% patella, 29% trochlea, 12% femoral condyle, and 8% tibial plateau)	Injection of peripheral blood-derived MSCs and HA weekly (×5) starting 1 wk after subchondral drilling and then weekly (×3) at 6 months; pre-injection GCSF	24 months	Arthroscopic: smooth defect filling. Histologic: ICRS II score was significantly better in MSC + HA group. MRI: improved cartilage morphology, defect filling and integration in MSC + HA group. Clinical: improvement in IKDC scores with no difference between MSC + HA and HA

ACI, autologous chondrocyte implantation; ACL-R, anterior cruciate ligament reconstruction; AOFAS, American Orthopaedic Foot and Ankle Society; BM, bone marrow; BMDC, bone marrow-derived cell; CMBMC, covered microfracture and bone marrow concentrate; CRA, Cartilage Repair Assessment (arthroscopy); F, female; GCSF, granulocyte colony stimulating factor; HA, hyaluronic acid; HTO, high tibial osteotomy; ICRS, International Cartilage Repair Society; IKDC, International Knee Documentation Committee; KOOS, Knee Injury and Osteoarthritis Outcome Score; M, male; MACI, matrix-associated autologous chondrocyte implantation; MFX, microfracture; MRI, magnetic resonance imaging; MSC, mesenchymal stem cell; OA, osteoarthritis; OCD, osteochondral dissecans; PGA-HA, polyglycolic acid-hyaluronic acid; RCT, randomized controlled trial; RHSSK, Revised Hospital for Special Surgery knee; SF-36, Short Form-36; VAS, Visual Analogue Scale; wk, week(s); y, year(s).

Following aspiration, MSCs were isolated and expanded within the laboratory for 2 to 3 weeks and implanted alone [84,93] or in association with biomaterial matrices [82,83,85,88]. Alternatively, in other studies, BMDCs - also described as bone marrow concentrate [86,87] or bone marrow aspirate concentrate [89] - were separated using centrifugation systems [80,81,86,87,89–91]. Presumably, these collections contained a variety of cell types from the bone marrow space, some of which were MSCs. BMDCs were immediately implanted in conjunction with matrices into defects in the same operative period as the aspiration. Matrices used in these studies included platelet-rich fibrin gel [80,81,83,89,90], fibrin glue [86,87], collagen gel and paste [80,82,88,90,91], and scaffolds composed of collagen [82,85,86,88,89], HA [80,81,90–92], and PGA-HA [87]. In most cases, MSCs or BMDCs were seeded onto scaffold or gel matrices for implantation. Combinations of scaffolds and cell-containing gels or glue were commonly described [80–82,86–88,90–92,96]. Some protocols involved the implantation of cells within gel followed by coverage with biomaterial membranes [86,87,89]. Cell-matrix constructs were implanted on the same day of scaffold seeding [80,81,90–92,96] or following a few days of *in vitro* culture in an attempt to promote cell adherence to scaffolds prior to implantation [82,88]. Some groups used fibrin glue [84] and overlying periosteal flaps [82–84,88,93,95] or synovium [88] to stabilize implanted constructs.

Based on the available early evidence, implantation of MSCs or BMDC collections containing MSCs appears to be a successful treatment for focal traumatic chondral and osteochondral defects (Table 3). Clinical outcomes improved with time over 24 months following implantation in the majority of patients with focal chondral and osteochondral lesions of the knee [81,84–89] and ankle [80,90,91]. These positive outcomes contrast with those from patients with more advanced degenerative disease. In one study focusing on the management of unicompartmental OA of the knee, outcomes were equivalent between the MSC transplantation group and cell-free control group in 24 patients who underwent concomitant high tibial osteotomy [95]. Furthermore, one participant with OA in another case series had worse clinical scores post-operatively than others with focal defects [83].

**Table 3:** **Current mesenchymal stem cell transplantation protocols**

**Construct**	**Transplantation protocol**	**Advantages**	**Disadvantages**
**BMDC-seeded scaffold** [75,80,81,89–91]	Bone marrow aspiration, separation of nucleated cell population (BMDCs) by centrifugation, scaffold seeding, and implantation of BMDC-scaffold construct into the AC defect site	Accessory cells/GFs create a natural microenvironment	Low number of MSCs
		One step procedure with aspiration and implantation in the same surgery	Cells other than MSCs could promote immunorejection in allogeneic transplantation
**MSC-seeded scaffold** [38,65,72,82–85,88]	Bone marrow aspiration, *in vitro* MSC isolation by adherence to plastic flasks, *in vitro* expansion of MSCs, scaffold seeding with MSCs, and implantation of MSC-scaffold construct into the AC defect site	High MSC numbers are available due to expansion	*In vitro* expansion may increase the risk of contamination
		Isolation allows for purification of MSCs and potentially reduced likelihood of rejection in allogeneic transplant	MSCs have the capacity to become bone without *in vitro* cueing prior to implantation (bone may be beneficial in osteochondral lesions)
		Mid-range time consumption	
**Precultivated MSC-seeded scaffold** [38,66,72]	Bone marrow aspiration, MSC isolation by adherence to plastic flasks, expansion of MSCs *in vitro*, scaffold seeding with MSCs, *in vitro* precultivation in medium promoting chondrogenesis, and implantation of a cartilage tissue construct into the AC defect site	High MSC numbers are available due to expansion	*In vitro* expansion and cultivation may increase the risk of contamination
		Chondrogenesis is stimulated	Highest time and resource consumption
		Increased mechanical stability of the implanted construct	No clinical assessment to date
		Early neo-tissue remodeling occurs *in vitro* and may be accounted for at the time of implantation	

AC, articular cartilage; BMDC, bone marrow-derived cell; GF, growth factor; MSC, mesenchymal stem cell.

To date, there is a relative paucity of literature assessing clinical outcomes beyond 24 months in patients treated with MSC or BMDC transplantation for focal AC defects. One group reported outcomes up to 48 months and noted a slight decrease in American Orthopaedic Foot and Ankle Society (AOFAS) scores at both 36 and 48 months compared with 24 months post-implantation [91]. Longer-term evidence is now available from Wakitani and colleagues [97] supporting the safety of MSC transplantation up to 137 months post-surgery, although other outcomes were not assessed.

MRI and arthroscopy have shown that repair tissue derived from MSC and BMDC transplantation contains hyaline-like cartilage and integrates within surrounding native tissue within 24 months of implantation [80,81,85–87,91] (Table 2). Cartilage quality correlated with clinical outcomes [81,91] as did implant-defect congruity and the amount of defect filling [83]. In some cases, hypertrophic cartilage has been noted on arthroscopy, but healthy repair tissue was found upon arthroscopic debridement of this tissue [80]. Lack of complete filling and non-congruent resurfacing of defects have been reported in a minority of cases [83]. In osteochondral lesions, subchondral bone appears to require longer periods of time than cartilage for remodeling. Giannini and colleagues [91] found abnormal subchondral structure and separated osteochondral interfaces on MRI at 24 months following treatment of osteochondral lesions of the talus.

Histological analysis of repair tissue biopsies has been consistent with MRI and arthroscopic findings [80–82,86,87,89] (Table 2). A number of groups have reported intense proteoglycan staining surrounding differentiated chondrocytes [80,81,87,89]. Furthermore, repair tissue often contained a moderate to large amount of collagen II with lesser amounts of collagen I on immunohistochemistry that supported the presence of hyaline-like cartilage phenotype [80,82,84,89]. Fibrocartilage or mixed repair tissue has also been described, but in a relatively smaller number of patients [82,87,88]. Periosteal flaps and subchondral drilling were used in these studies and are potential contributing factors.

Two clinical studies have compared MSC/BMDC transplantation to chondrocyte transplantation (ACI/MACI) [84,90]. Similar positive outcomes were noted on most clinical scales. Better physical role functioning on the ICRS Package Short Form-36 (SF-36) scale was noted with MSCs relative to chondrocytes [84]. MRI, arthroscopic and histologic findings indicated that both procedures were capable of resurfacing defects with hyaline-like cartilage repair tissue that integrated into surrounding cartilage [84,90].

Although implantation of MSC-based constructs has been the focus of clinical literature to date, one group has reported outcomes following intra-articular injection of MSCs for the treatment of focal chondral defects [29,98]. In a randomized controlled trial, autologous peripheral blood MSCs were injected with HA weekly for 5 weeks after subchondral drilling and subsequently for another 3 weeks at 6 months into the knees of patients with lesions of the femoral condyle, tibial plateau, patella, and femoral trochlea. Histologic assessment at 18 months showed the presence of hyaline-like cartilage in patients who received MSCs. Furthermore, ICRS II histologic scores were significantly better in participants who received MSCs and HA versus those who received HA. However, International Knee Documentation Committee (IKDC) clinical scores were equivalent between these two groups at 24 months.

## Optimizing technique in mesenchymal stem cell transplantation

**Figure 2:**
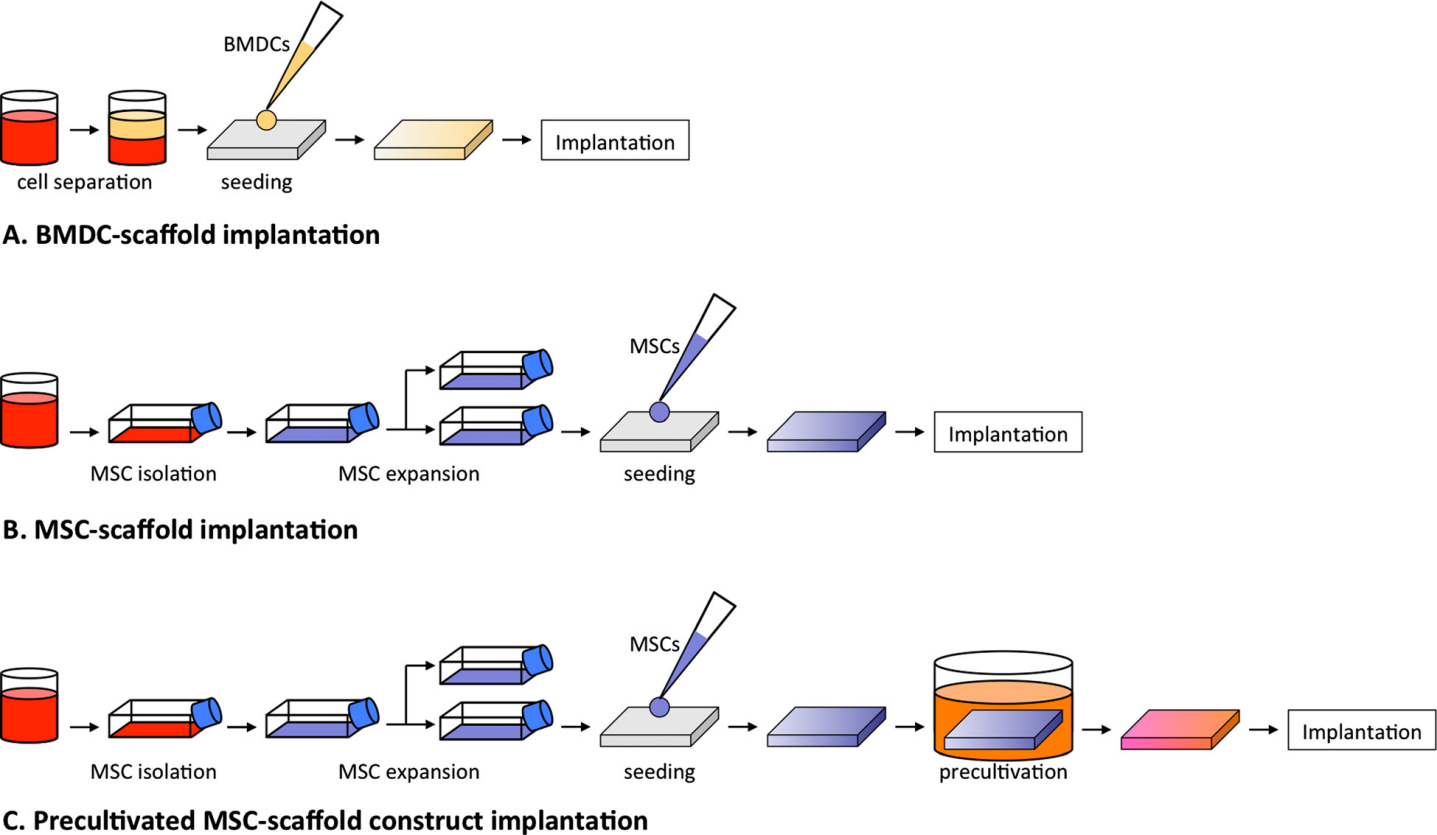
**Mesenchymal stem cell transplantation constructs and protocols. (A)** In bone marrow-derived cell (BMDC) transplantation, the bone marrow aspirate is centrifuged to create a BMDC concentrate that contains mesenchymal stem cells (MSCs) within a pool of other cells and chemical mediators. BMDCs are then seeded onto a scaffold and implanted within a cartilage defect. **(B,C)** MSC transplantation involves isolating MSCs from a bone marrow aspirate by plastic adherence and expansion in plastic tissue culture flasks. MSCs are then seeded onto a scaffold and implanted **(B)** or precultivated *in vitro* to promote chondrogenesis prior to implantation **(C)**.

The goal of MSC transplantation is to create repair tissue with properties of hyaline cartilage that integrates into surrounding native osteochondral tissue while limiting local and systemic adverse effects. Three general MSC transplantation protocols currently exist (Figure 2). The one-step BMDC transplantation protocol consists of bone marrow aspiration, separation of a nucleated cell population containing MSCs amongst other cells, seeding of these cells on a scaffold, and implantation all within a single operative period [80,81,89–91]. A second protocol involves isolation of MSCs within the laboratory, *in vitro* expansion, and scaffold seeding shortly before implantation [82–85,88]. The scaffold may be seeded at the time of implantation or within a few days after a short *in vitro* culture period to promote MSC adherence to the biomaterial [82]. The final protocol utilizes isolated, expanded MSCs that are seeded onto a scaffold and precultivated - or pre-differentiated - *in vitro* over 2 to 3 weeks to promote chondrogenesis prior to implantation [38,66,72].

BMDC transplantation and non-precultivated, isolated and expanded MSC transplantation have both resulted in the creation of hyaline-like cartilage based on arthroscopy, histology and imaging, and yielded positive outcomes in clinical studies [80,82–85,88–91]. To our knowledge, implantation of precultivated MSC-matrix constructs has not been studied clinically to date, but was shown to produce hyaline-like cartilage tissue in large animal *in vivo* studies [38,66,72]. Although successful resurfacing has been performed with all three transplantation protocols, each carries specific advantages and disadvantages that are described in Table 3.

At present, studies comparing these protocols in humans are lacking, but have been performed in animal models. Zhang and colleagues [75] found no difference at 2 months between BMDCs and expanded MSCs seeded on collagen gels implanted within femoral trochlea chondral defects in mini-pigs. Marquass *et al.* [38,72] showed that precultivated MSC-seeded collagen gels produced superior repair tissue after 1 year compared with non-precultivated MSCs within distal femur osteochondral defects in sheep.

Regardless of the specific transplantation protocol used, MSC-based therapies require a number of steps that may be optimized to improve MSC yield, chondrogenesis, repair tissue integration, and ultimately clinical outcome. These steps may include cell collection, MSC isolation and expansion, matrix seeding, precultivation into cartilage tissue, and surgical implantation.

### Collection of mesenchymal stem cell-containing tissue

MSCs are present within a number of tissues that may serve as potential harvest sites (Table 4). To date, needle aspiration of pelvic bone marrow has been the method of choice for MSC-based treatment of human AC defects [80,81,83–85,87–91,99]. The use of peripheral blood MSCs has also been reported clinically [29,98]. Synovial, periosteal and adipose tissues are other sources that have been assessed *in vivo* in the animal literature with relevance [7,53,70]. Synovial MSCs appear to have improved chondrogenic capacity relative to MSCs from bone marrow and periosteum based on *in vitro* assessment [28], although this advantage has not been reproduced *in vivo* in two rabbit studies [53,56]. Adipose tissue offers the advantage of abundant availability, but adipose MSCs have a lower chondrogenic potential than MSCs from bone marrow, synovium and periosteum [28]. Bone marrow- and periosteum-derived MSCs have a heightened osteogenic potential [28]. Although this may be not ideal for cartilage engineering, it could be advantageous in osseous regeneration within osteochondral lesions. While present-day techniques utilize autologous MSCs in transplantation, MSC tissue banking and allogeneic transplantation could one day provide an alternative strategy [100]. However, further investigation is required to support allogeneic use of MSCs due to recent work suggesting that both undifferentiated and chondrogenic-differentiated MSCs cause immunoreaction [101].

**Table 4:** **Clinically relevant sources of mesenchymal stem cells for cartilage engineering**

**MSC source**	***In vivo*** **assessment of focal AC defect treatment**	**Advantages**	**Disadvantages**
**Bone marrow**	Clinical and pre-clinical [7,38,45,47,48,51,53,59,60,65,66,68,69,71–73,76–78,80–85,88–91,97]	Most rigorous investigation and strongest supporting evidence	Propensity to form osseous tissue (could be beneficial for osseous regeneration in osteochondral lesions)
		Ease of collection by needle	
		Long-term safety reported	
**Peripheral blood**	Clinical and pre-clinical [29,61,98]	Ease of collection by needle	Paucity of literature comparing this source to others
**Synovial tissue**	Pre-clinical [50,52–54,56,62,64,67,70]	Greatest chondrogenic capacity noted based on *in vitro* study	Clinical assessment is lacking
**Periosteum**	Pre-clinical [7,56]	Equivalent chondrogenic capacity to bone marrow	Propensity to form osseous tissue
			Clinical assessment is lacking
**Adipose tissue**	Pre-clinical [46,49,56]	Abundance of tissue	Reduced chondrogenic capacity
		Widespread anatomic availability	Clinical assessment is lacking

AC, articular cartilage; MSC, mesenchymal stem cell.

### Isolation and expansion of mesenchymal stem cells

Following bone marrow collection, tissue is placed in serum-containing medium within plastic culture flasks and incubated for a number of days [26]. Mononucleated cells, some of which are MSCs, may be quantified and plated at 10,000 cells per cm^2^ [84]. MSC isolation occurs through adherence of MSCs to plastic, as other cell types are non-adherent and discarded when culture medium is changed. Cell replication is monitored through the level of confluence observed by microscopy. Once confluence is achieved, trypsin and EDTA are used to disrupt adherence, and MSCs are re-plated within a larger number of flasks [30]. This process is repeated through multiple passages to allow for expansion to occur. Although a greater number of passages yields a larger number of total MSCs available for implantation, proliferation and chondrogenic differentiation potential decrease and may be lost if cells are expanded through too many passages [102,103]. These cells are then destined to undergo osteogenesis [104]. As a result, MSCs are usually expanded through a maximum of two to three passages.

Given that the ratio of MSCs to other cells within the bone marrow is estimated at one in 10,000 cells, expansion is beneficial and must be optimized to ensure that an adequate yield of pure MSCs is available for implantation [105]. Basic fibroblast growth factor (FGF-2 or bFGF) within culture medium increases MSC growth rate and maintains multipotency [106]. Furthermore, FGF-2 has been shown to increase collagen and proteoglycan gene expression and GAG production [107,108]. Hypoxia during MSC expansion also augments chondrogenic potential [109]. GAG synthesis and gene expression of collagen II and Sry-related HMG box (SOX)9 significantly increased in MSC pellet cultures expanded under 3% O_2_ compared with those expanded under 21% O_2_ [109]. Bioreactors may also be used to improve yield; these provide efficient nutrient exchange and allow for increased cell densities during expansion [105].

### Biomaterial matrix selection and seeding

Biomaterial matrices provide a framework for MSC proliferation and differentiation, and consolidate MSCs into three-dimensional structures capable of filling defects. The vast majority of pre-clinical and clinical studies to date have used matrices. Gels or pastes composed of collagen or platelet-rich fibrin are moldable substances that hold the cells suspended [80,82]. Porous scaffolds made of materials such as collagen and HA serve as malleable, foam-like structures that adhere MSCs at the time of seeding [66,82]. Cell-seeded scaffolds with multiple layers engineered for osteochondral lesions have shown positive results in animal studies [47]. A tri-layer scaffold composed of collagen and hydroxyapatite has been tested in humans, but was used as a cell-free scaffold without co-implantation of MSCs [110]. There is potential for use of this product in conjunction with MSCs in the future. Combinations of MSC-embedded gels and scaffolds have been used in some *in vivo* studies [80,87,91,95].

MSC seeding density has not been routinely reported to date in clinical studies. One reason for this is that some trials have used BMDCs, only a few of which are MSCs, rather than pure isolated MSCs [92]. In the *in vivo* animal literature, MSC densities of 10 to 48 million cells per cm^3^ of scaffold have been used [45,47,66]. The optimal number of MSCs to be seeded per unit volume is currently unknown.

### Cell-seeded biomaterial matrix implantation and reinforcement

Standard open or arthroscopic surgical approaches to the knee or ankle are used to access chondral and osteochondral defects during implantation procedures [80,82,92,96]. Damaged AC is debrided down to subchondral bone and the edges are trimmed until a rim of healthy AC is evident [82,84,93]. In the setting of full-thickness chondral defects, drilling of intact subchondral bone has been used by some groups in an attempt to stimulate the influx of cells and mediators into the repair zone [83,86–88,95], while other groups have left subchondral bone intact [82,93,111]. Subchondral bone is already disrupted in osteochondral lesions. Therefore, careful debridement of malacic bone may be performed until healthy bone is reached [80].

At the time of implantation, matrices or cell-matrix constructs may be resized with punches or scalpels to fit within the margins of the defect [80]. Implantation orientation may be relevant in scaffolds engineered with separate porous and non-porous sides [45,95].

Fibrin glue or autologous platelet-rich fibrin gel may be deposited within the defect and overlying the MSC-matrix construct to augment implant fixation [45,83,84,92]. Sutures may be used to anchor cell-seeded scaffolds to surrounding native cartilage [111]. Autologous periosteal flaps or biomaterial membranes have also been used to prevent leakage of MSCs from cell collections implanted alone [84,93] or embedded within collagen or fibrin gel [82,83,86,87,95]. Periosteal flaps have been shown to form superficial fibrous caps that cover hyaline cartilage repair tissue [82]. In general, they are not recommended for use in scaffold-associated cell-based therapies, but may be used to contain implanted MSCs within defect areas when scaffolds are not used [84].

### *In vitro* precultivation of bioengineered tissue

Various *in vitro* culture techniques have been elucidated that may be used to promote the creation of hyaline cartilage within precultivated MSC-scaffold constructs. Chemical mediators such as TGF-β and dexamethasone are placed with culture media for chondrogenic induction [30]. Several factors, including bone morphogenetic proteins (BMP-2, −4 and −6) and insulin-like growth factor (IGF)-1 may be used in addition to TGF-β and dexamethasone to amplify chondrogenesis [42,112]. Ascorbic acid serves as a cofactor in the hydroxylation of amino acids in collagen, and is also routinely used within chondrogenic culture medium [113].

Incubator oxygen tension during precultivation may be used to modulate chondrogenesis. Hypoxic exposure was found to increase ECM deposition on scaffolds and gene expression of collagen II, aggrecan and Sox9 in pellet cultures [114]. Co-culture of MSCs with chondrocytes promotes the creation of cartilage through chondrocyte-enhanced MSC chondrogenesis [115]. Pellet co-culture of human MSCs and chondrocytes increased GAG deposition and expression of type II collagen while enhancing MSC-induced chondrocyte proliferation [115]. Cartilage formation may be augmented by mechanical stimulation during cultivation through either hydrostatic pressure or ultrasound. Hydrostatic pressure in constant and cyclic forms increased sulfated GAG matrix deposition by chondrocytes cultured on collagen scaffolds [116]. Furthermore, low-intensity ultrasound improved histological chondrogenic morphology, GAG and collagen II content, as well as gene expression of type II collagen, aggrecan and SOX9 [37,117].

Bone marrow-derived MSCs have the propensity to enter an osteogenic pathway, a property that is not ideal for AC engineering [104]. During precultivation, osteogenesis may be dampened using a variety of methods. Parathyroid hormone-related protein has been shown to reduce collagen X gene expression and alkaline phosphatase activity [118]. Hypoxic culture of MSCs significantly suppressed expression of collagen X relative to normoxic culture [109]. Lastly, co-culture of MSCs with chondrocytes reduced osteogenesis based on osteocalcin quantification, and Von Kossa and Alizarin Red staining [119].

## Discussion: current recommendations and future directions

MSC-based therapy through injection or implantation is a promising treatment for traumatic chondral and osteochondral defects. MSC injection offers the advantage of minimal invasiveness, but dispersion of injected MSCs and lack of focus of these cells into defects make this method less appealing than direct implantation techniques. Several pre-clinical studies have been performed, but only one group has assessed MSC injection clinically to date [29]. The current literature supports performing microfracture or subchondral drilling in conjunction with weekly injections of MSCs and HA over the course of multiple weeks [29,69,78]. This protocol presumably increases the likelihood of defect seeding with MSCs from both injection and subchondral marrow sources.

MSCs may be implanted alone or in conjunction with a biomaterial matrix. MSCs implanted and covered with a periosteal flap in a procedure analogous to ACI produced good outcomes based on one clinical study [84]. The majority of the current clinical and pre-clinical literature has focused on matrix-associated transplantation of MSCs. Three general construct types have been implanted, including biomaterial scaffolds seeded with either BMDCs [80] or isolated and expanded MSCs [82], and precultivated constructs derived from MSCs cultured *in vitro* on scaffolds prior to implantation [38]. All three protocols are capable of resurfacing focal AC defects with hyaline-like cartilage that integrates with surrounding tissue [38,82,89], while each has unique advantages and disadvantages. At present, all may be considered as potential treatment options. BMDC-scaffold implantation and non-precultivated isolated, expanded MSC-scaffold implantation have led to positive functional, arthroscopic, histologic and radiographic outcomes at 12 to 48 months in patients with traumatic, focal chondral and osteochondral defects of the knee and ankle [80–84,88,89]. No clinical studies have compared these two protocols, although one preclinical study found equivalent histologic outcomes [75]. The third protocol, precultivated MSC-scaffold construct implantation, has only been investigated in pre-clinical models but should be considered for clinical implementation given that it produced superior repair tissue in comparison to non-precultivated MSC-scaffold constructs over 6 to 12 months in two sheep studies [38,72].

It is currently unclear whether defect characteristics should dictate the transplantation protocol used. Both full-thickness chondral and osteochondral defects have been treated successfully with the current modalities, but several important differences exist between these defect types. In the setting of full-thickness chondral lesions, the subchondral bone is intact and there is no diffusion of subchondral marrow contents (MSCs, accessory cells and growth factors) into the defect site. Some groups have therefore recommended bone marrow stimulation techniques, such as microfracture and subchondral drilling, to be performed as an adjunct to MSC/BMDC-scaffold implantation [88,99]. However, other groups have not used these techniques [80,81,84,90,91], possibly because the repair tissue derived from microfracture or subchondral drilling may produce fibrocartilage that is mechanically inferior to hyaline cartilage [9]. However, histologic assessment following combined arthroscopic microfracture and BMDC transplantation - described as the covered microfracture and bone marrow concentrate technique by Gigante and colleagues [86,87] - showed the presence of hyaline-like cartilage tissue.

Osteochondral defects are deeper defects that involve subchondral plate disruption and diffusion of subchondral marrow contents into the defect site. These defects require more complex regeneration of separate layers of cartilage and bone. Tissue consistent with hyaline cartilage has been found following MSC transplantation [80,81,91]. Subchondral bone regeneration has also been reported, but restoration requires an extended period of time relative to cartilage. Specifically, 29 months following treatment of femoral condyle osteochondral defects, Buda and colleagues [81] noted cartilage surface intactness in 70%, iso-intense cartilage tissue in 65%, but subchondral lamina and bone intactness in only 30% of participants on MRI. Similarly, at 24 months following treatment of talus osteochondral defects, Giannini and colleagues [91] reported cartilage surface intactness in 40%, iso-intense cartilage tissue in 70%, but subchondral lamina and bone intactness in only 10% and 35%, respectively.

Detailed comparisons between MSC transplantation and other modalities of treatment for traumatic AC defects are lacking in the current literature. Chondrocyte transplantation (ACI/MACI), the current gold standard of cell-based treatment, has shown positive outcomes up to 10 to 20 years [20], while MSC/BMDC transplantation has only been assessed for up to 2 to 4 years. In our review, one pre-clinical large animal study and two clinical studies were found that directly compared MSC transplantation to chondrocyte transplantation [72,84,90]. Marquass and colleagues [72] reported superior histologic findings in repair tissue derived from precultivated MSCs in comparison to chondrocytes at 1 year post-implantation in sheep. Clinically, Nejadnik and colleagues [84] found similar positive functional outcomes on IKDC, Tegner and Lysholm scales between MSC implantation and ACI in the treatment of knee defects, but noted significantly higher physical role functioning on the ICRS Package SF-36 in patients treated with MSCs. Giannini and colleagues reported similarly improved AOFAS scores with both MSC and chondrocyte transplantation following treatment of talus defects [90]. Further comparative evaluation is required to better assess MSC transplantation relative to chondrocyte implantation.

MSC transplantation may reduce the likelihood of low chondrocyte yield and chondrocyte de-differentiation associated with chondrocyte transplantation [23,25,103]. Chondrocyte senescence is a concern with ACI/MACI [24,120,121], and, although MSCs also undergo senescence over prolonged periods of proliferation, adequate MSC yields for transplantation may be attained at a stage during which there is still significant residual proliferative capacity [104]. Chondrocyte transplantation requires two invasive surgical procedures, one for cell collection and one for implantation [20]. In contrast, MSC transplantation only requires one surgical procedure at the time of implantation [88]. MSC collection may be performed through minimally invasive needle aspiration [82]. Consequently, MSC transplantation may be less expensive than ACI/MACI. Giannini and colleagues [90] found that the total cost of arthroscopic matrix-associated BMDC transplantation was half of the cost of arthroscopic MACI and one-third of the cost of open ACI.

Several technical steps may be optimized in MSC transplantation to promote cell numbers, chondrogenesis, repair tissue integration, and clinical outcome. With respect to cell collection, autologous bone marrow has been used in all clinical studies to date and is the current preference [80–85,88–91]. However, synovial MSCs appear to have superior chondrogenic capacity and should be considered [28]. Furthermore, adipose tissue is abundantly available [49]. In MSC isolation, plastic adherence is used [30]. Expansion may be augmented using serum-containing medium supplemented with FGF-2 and incubation under hypoxic conditions [106,109]. Several matrix types are appropriate for MSC transplantation. At present, three-dimensional scaffolds composed of collagen or hyaluronic acid are the standard [81,82]. Scaffolds composed of multiple layers are an option in the setting of osteochondral lesions [47]. Precultivation of MSC-scaffold constructs should be performed in serum-free medium containing TGF-β and dexamethasone supplemented with other mediators such as ascorbic acid, IGF-1 or BMPs [30,42,112]. Hypoxic incubation, co-culture with chondrocytes, mechanical stimulation with ultrasound, and dynamic culture within a bioreactor are other precultivation optimizing techniques that should be considered [37,105,109,115]. Implantation may be performed through either open or arthroscopic techniques. Fibrin glue or autologous platelet-rich fibrin gel may be used to stabilize implanted constructs [83,85]. At present, there is insufficient evidence to support the use of marrow stimulation (that is, subchondral drilling or microfracture) or periosteal flaps in MSC transplantation.

## Conclusion

MSC transplantation is a promising cell-based strategy for the treatment of traumatic chondral and osteochondral defects of the knee and ankle. Although there is currently no established consensus protocol, multiple technical variations have successfully produced hyaline-like cartilage repair tissue that integrates within native tissue. Duplication and optimization of current protocols are important to improve the cartilage ECM formed in a reliable and safe manner. Clinical studies to date report positive outcomes at 12 to 48 months following MSC implantation. Future investigation will provide insight into long-term outcomes relative to other treatment modalities and clarify whether MSC transplantation may replace present-day techniques as the gold standard.

## Additional file

Additional file 1:
**MEDLINE, EMBASE and Web of Science database search strategies.**

## Abbreviations

AC: Articular cartilage
ACI: Autologous chondrocyte implantation
AOFAS: American Orthopaedic Foot and Ankle Society
BMDC: Bone marrow-derived cell
BMP: Bone morphogenetic protein
ECM: Extracellular matrix
EDTA: Ethylenediamine-tetraacetic acid
FGF: Fibroblast growth factor
GAG: Glycosaminoglycan
HA: Hyaluronic acid
ICRS: International Cartilage Repair Society
IGF: Insulin-like growth factor
IKDC: International Knee Documentation Committee
MACI: Matrix-associated autologous chondrocyte implantation
MRI: Magnetic resonance imaging
MSC: Mesenchymal stem cell
OA: Osteoarthritis
PGA: Polyglycolic acid
PLA: Polylactic acid
PLGA: Polylacticglycolic acid or polylactide co-glycolide
SF-36: Short Form-36
TEC: Tissue-engineered construct
TGF-β: Transforming growth factor-beta
