# Supplementary material for: Mesenchymal stem cells in the treatment of traumatic articular cartilage defects: a comprehensive review
Source: Arthritis Res Ther. 2014 Sep 26;16:432. doi: 10.1186/s13075-014-0432-1 (PMC4289291; doi:10.1186/s13075-014-0432-1)
Supplement: Additional file 1: — MEDLINE, EMBASE and Web of Science database search strategies. [file 13075_2014_432_MOESM1_ESM.pdf]

MEDLINE search strategy

Ovid MEDLINE(R) In-Process & Other Non-Indexed Citations, Ovid MEDLINE(R) Daily and Ovid MEDLINE(R) 1946 to Present

Search steps

Limits: English, 1994-current

1. (cartilag* or chondrogen*).ti,ab.
2. cartilage/ or cartilage, articular/
3. Chondrogenesis/
4. osteochondr*.ti.
5. or/1-4
6. exp Mesenchymal Stem Cell Transplantation/ or exp Mesenchymal Stromal Cells/
7. Stromal Cells/
8. ((mesenchymal adj3 cell*) or (bone marrow adj2 cell*) or (bone marrow and stromal cell*)).ti,ab.
9. or/6-8;
10. 5 and 9
11. 10 not osteoarthritis.ti.
12. limit 11 to (english language and yr="1994-Current")
13. In Vitro/
14. (monolayer* or cell aggregat* or pellet* or micromass* or transwell* or hydrogel* or "ex vivo" or explant*).ti,ab.
15. in vitro.ti.
16. or/13-15
17. 12 and 16
18. in vivo.ti,ab.
19. 17 and 18
20. 12 not 17
21. humans/ not (animals/ and humans/)
22. 20 not 21
23. 19 or 22
24. 12 not (17 or 22)

Output

In vitro articles: search step 17
In vivo animal articles: search step 23
Clinical articles: search step 24

EMBASE search strategy

Embase 1974 to 2014 July 02

Search steps

Limits: English, 1994-current, articles not within MEDLINE (previous search)

1. (cartilag* or chondrogen*).ti,ab.
2. cartilage/ or cartilage, articular/
3. Chondrogenesis/
4. osteochondr*.ti.
5. or/1-4
6. exp Mesenchymal Stem Cell Transplantation/ or exp Mesenchymal Stromal Cells/
7. Stromal Cells/
8. ((mesenchymal adj3 cell*) or (bone marrow adj2 cell*) or (bone marrow and stromal cell*)).ti,ab.
9. or/6-8
10. 5 and 9
11. 10 not osteoarthritis.ti.
12. limit 11 to (english language and yr="1994-Current")
13. in vitro study/ or exp vivo study/
14. (monolayer* or cell aggregat* or pellet* or micromass* or transwell* or hydrogel* or "ex vivo" or explant*).ti,ab.
15. in vitro.ti.
16. or/13-15
17. 12 and 16
18. in vivo.ti,ab.
19. 17 and 18
20. 12 not 17
21. humans/ not (animals/ and humans/)
22. 20 not 21
23. 19 or 22
24. 12 not (17 or 22)
25. in vivo study/
26. 12 and 25
27. 23 or 26
28. limit 17 to exclude medline journals
29. limit 27 to exclude medline journals
30. limit 24 to exclude medline journals

Output

In vitro articles: search step 28
In vivo animal articles: search step 29
Clinical articles: search step 30

Web of Science search strategy

Version 5.13.3

Search steps

Limits: 1994-2014, English, Article

1. TOPIC: ((mesenchymal NEAR/3 cell*) OR ("bone marrow" NEAR/2 cell*)) AND (cartilage OR chondrogen*)
2. TITLE: (meniscus OR osteoarthitis)
3. #1 NOT #2
4. TOPIC: ("in vitro" OR monolayer* OR cell aggregat* OR pellet* OR micromass* OR transwell* OR hydrogel* OR "ex vivo" OR explant*)
5. #3 AND #4
6. TOPIC: (transplant* OR defect* OR lesion*) OR “in vivo”
7. TOPIC: (animal OR rat OR rabbit OR dog OR sheep OR horse OR pig OR goat OR murine OR leporine OR canine OR ovine OR equine OR porcine OR caprine)
8. #6 AND #7
9. (#3 AND #8) NOT #5
10. TOPIC: (human OR epidemiologic* OR observational OR "follow up" OR cohort OR retrospective OR "case series" OR "case report" OR "case study" OR "transplant*" OR "defect" OR "lesion")
11. (#3 AND #10) NOT (#5 OR #9)

Output

In vitro articles: search step 5
In vivo animal articles: search step 9
Clinical articles: search step 11
